# Restricting tumor lactic acid metabolism using dichloroacetate improves T cell functions

**DOI:** 10.1186/s12885-021-09151-2

**Published:** 2022-01-06

**Authors:** Hosein Rostamian, Mohammad Khakpoor-Koosheh, Leila Jafarzadeh, Elham Masoumi, Keyvan Fallah-Mehrjardi, Mohammad Javad Tavassolifar, John M. Pawelek, Hamid Reza Mirzaei, Jamshid Hadjati

**Affiliations:** 1 Department of Medical Immunology, School of Medicine, Tehran University of Medical Sciences, Tehran, Iran; 2 Department of Laboratory Sciences, Sirjan School of Medical Sciences, Sirjan, Iran; 3 Department of Immunology, School of Medicine, Ilam University of Medical Sciences, Ilam, Iran; 4 Department of Dermatology and the Yale Cancer Center, Yale School of Medicine, New Haven, CT USA

**Keywords:** Metabolism, Lactic acid, Cancer, T cell, Dichloroacetate, Immunotherapy

## Abstract

**Background:**

Lactic acid produced by tumors has been shown to overcome immune surveillance, by suppressing the activation and function of T cells in the tumor microenvironment. The strategies employed to impair tumor cell glycolysis could improve immunosurveillance and tumor growth regulation. Dichloroacetate (DCA) limits the tumor-derived lactic acid by altering the cancer cell metabolism.

In this study, the effects of lactic acid on the activation and function of T cells, were analyzed by assessing T cell proliferation, cytokine production and the cellular redox state of T cells. We examined the redox system in T cells by analyzing the intracellular level of reactive oxygen species (ROS), superoxide and glutathione and gene expression of some proteins that have a role in the redox system. Then we co-cultured DCA-treated tumor cells with T cells to examine the effect of reduced tumor-derived lactic acid on proliferative response, cytokine secretion and viability of T cells.

**Result:**

We found that lactic acid could dampen T cell function through suppression of T cell proliferation and cytokine production as well as restrain the redox system of T cells by decreasing the production of oxidant and antioxidant molecules. DCA decreased the concentration of tumor lactic acid by manipulating glucose metabolism in tumor cells. This led to increases in T cell proliferation and cytokine production and also rescued the T cells from apoptosis.

**Conclusion:**

Taken together, our results suggest accumulation of lactic acid in the tumor microenvironment restricts T cell responses and could prevent the success of T cell therapy. DCA supports anti-tumor responses of T cells by metabolic reprogramming of tumor cells.

**Supplementary Information:**

The online version contains supplementary material available at 10.1186/s12885-021-09151-2.

## Introduction

Cancer immunotherapy through adoptive cellular therapy (ACT) and its derivative, chimeric antigen receptor (CAR) T cells, has shown clinical effectiveness for hematological malignancies and immunogenic tumors such as melanoma but the efficacy of ACT for solid tumors to date has been limited and challenges still remain. This may in part be due to the microenvironment immunosuppressive nature of tumor cells [1–3]. Metabolites produced by the cancer cells can inhibit T cells in the tumor microenvironment, e.g. high concentrations of lactic acid and extracellular acidosis are typical features of tumors [4, 5].

Lactic acid accumulation in tumors is a by-product of hypoxia which occurs when tumors switch to an anaerobic metabolism. Also in the presence of oxygen some tumors undergo glycolysis, a phenomenon called the Warburg Effect [6]. Acidification by lactic acid promotes angiogenesis, immunosuppression and metastasis all of which are associated with poorer clinical outcome [7]. Lactic acid produced by highly glycolytic tumors has been shown to overcome immune surveillance by suppressing activation of NK and infiltrating T cells and inhibiting the proliferation and cytokine production of T lymphocytes [8, 9]. Some studies have shown that lactate has a relation with the reactive oxygen species (ROS) system. Lactate causes an increase in ROS production [10]. High levels of ROS can be cytotoxic agents because of their capability of destroying DNA and other subcellular structures, and antioxidants molecules keep ROS under strict control to avoid cellular damage. On the other hand, ROS were shown to be crucial second messengers for signaling of T cell receptor and T cell activation and T-cell redox regulation changes may influence the pathophysiology of a variety of human disorders [11, 12]. Superoxide dismutase (SOD) and catalase (CAT), which are regulated by Nrf2 are known as enzymatic elements and glutathione (GSH) is referred as non-enzymatic element of antioxidant system [13]. ROS are produced by activated T cells, which stimulate the antioxidative glutathione (GSH) response to protect cells from damage [14]. Strategies to impairment of Tumor cell glycolysis could improve immunosurveillance and tumor growth regulation [15]. and manipulation of the enzymes involved in tumor cell glycolysis might be a way to overcome immunosuppression [16]. Pyruvate Dehydrogenase Kinase (PDK) is a gatekeeper enzyme regulating metabolism of glucose in tumors. PDK inactivates the pyruvate dehydrogenase complex (PDC) through its phosphorylation. PDC converts pyruvate to acetyl-CoA, which is further metabolized in the mitochondria. Overexpression of PDK has been reported in several tumors and is associated with invasion, metastasis and chemotherapy drug resistance. High PDK expression contributes to a change in glucose metabolism towards glycolysis rather than oxidative phosphorylation [17]. Thus, PDK inhibition with the drug dichloroacetate (DCA) changes the cancer cell metabolism from glycolysis towards mitochondrial glucose oxidation and as a result reduces lactic acid levels [18].

In this study, we show that lactic acid can dampen T cell function through suppression of T cell proliferation, cytokine production, and TCR signaling. Lactic acid also suppressed the redox system of T cells and reduced production of both oxidant and antioxidant molecules. Our studies open new avenues to manipulate the metabolism of tumor cells by limiting tumor-derived lactic acid. Here, we tested the hypothesis of whether T cell function could be enhanced by pharmacological targeting of tumor glycolysis. DCA decreased the concentration of tumor lactic acid by suppression glucose metabolism of tumor cells leading to improvement of T cell function. T cell proliferation and cytokine production were increased in an in vitro co-culture test by pre-treating lymphoma cells with DCA. DCA also rescued the T cells from apoptosis. Therefore, DCA can overcome immunosuppression of lactic acid in the tumor microenvironment and could be useful for adoptive T cell immunotherapy.

## Methods

### Cell culture and media

The Raji cell line was acquired from the Iranian Biological Resource Center (IBRC). Cells were cultured In RPMI 1640 (Gibco, USA, cat. 21,875,034) with 10% Fetal bovine serum (FBS)(Gibco, cat. 11,573,397) and 1% penicillin/streptomycin (Sigma-Aldrich, USA) and incubated at 37 °C in 5% CO_2_. FBS was heat-inactivated for 30 min at 56 °C before use. CD19 expression on Raji cells was analyzed by flow cytometry utilizing APC-conjugated anti-human CD19 antibodies (Miltenyi Biotec, Germany) prior to the experiments. To assess pH of the media containing lactic acid, various concentrations of lactic acid (Sigma-Aldrich, L6661) were added to RPMI 1640 medium supplemented with 10% FBS and then measured with pH meter (figure S 1).

### PBMC isolation and T cell enrichment

Whole blood was taken from healthy donors. Using Ficoll–Paque density gradient centrifugation, human peripheral blood mononuclear cells (PBMCs) were isolated. Isolated PBMCs were seeded into 24-well plates (1.5 × 10^6^ cells/well) and cultured in RPMI1640 containing 10%FBS and 100 IU hIL-2 (Miltenyi Biotec). To activate and enrich T cells, PBMCs were cultured with 3 ug/ml anti-CD3 antibody (Miltenyi Biotec) and 10 ug/ml anti-CD28 antibody (Miltenyi Biotec). After 4 days of incubation at 37 °C, the purity of T cells was assayed using APC conjugated anti-human CD3 (BioLegend, USA) by flow cytometry. The clone of APC anti-human CD3 Antibody was UCHT1.The purity of T cells is represented in the figure S 2.

### Lactic acid production in tumor cell media

Raji cells were cultured in 1 ml complete media and were seeded into 24-well plates at 2 × 105 cells per well and They were treated with DCA (Alfa Aesar, USA) at 0.5 mM, 1 mM, 2 mM, 5 mM and 10 mM for 24 and 48 h. To measure lactate production the cell supernatants were harvested and lactate was measured using a colorimetric and lactate assay kit (Greiner Diagnostic GmbH, Germany). The cells viability were measured by flow cytometry using PI staining (figure S 3).

### T cell proliferation and cytokine assay

Raji cells were treated with mitomycin C (25 μg/ml) (Sigma-Aldrich) for 30 min to prevent proliferation [19]. Then mitomycin treated Raji cells (2 × 105 cells/well) were cultured in 24-well plates in the presence or absence of DCA (1 mM) for 24 h. The cell culture media containing DCA was removed and replenished with fresh RPMI 1640 complete media and they were co-cultured with CFSE-labeled T cells for 72 h. In order to label T cells with Carboxyfluorescein succinimidyl ester (CFSE) (Life Technologies, USA), the cells (1 × 10^6^ cells/well) were treated with 2.5 μM CFSE dye, and then 4 ml of FBS was applied to quench the reaction. CFSE-labeled T cells were co-cultured with Raji cells at 1:1 ratios (2 × 10^5^ cells/well) in RPMI1640 complete media without the presence of hIL-2 and anti-CD3/CD28 antibodies to induce the unspecific proliferation of T cells. T cells (2 × 10^5^ cells/well) also cultured without Raji cells in RPMI1640 complete media with and without lactic acid (20 mM) as a negative and positive control of lactic acid. T cells (2 × 10^5^ cells/well) were also cultured without anti-CD3/CD28 antibodies as unstimulated T cells. Cells were stained with anti-CD3-APC (BioLegend) and after 72 h, and CFSE dilution of CD3-gated lymphocytes was calculated by flow cytometry to evaluate their proliferation. T cell proliferation was determined based on the difference between the mean fluorescence intensity of stimulated and unstimulated cells. To measure the amount secreted IL-2 and IFN-γ by T cells, the supernatant was harvested 24 h after co-culture and assessed using ELISA kit (R&D Systems). For IL-2 and IFN-gamma detection, the ELISA kits utilized monoclonal Mouse IgG2A Clone # 5355 and monoclonal Mouse IgG2A Clone # K3.53, respectively.

### Apoptosis assay, Annexin V staining

Raji cells (2 × 10^5^ cells/well) were cultured in 24-well plates in the presence and absence of DCA (1 mM) for 24 h. Then the cell culture media containing DCA was removed and DCA-treated cells were co-cultured with T cells at 1:1 ratios (2 × 10^5^ cells/well) for 48 h. T cells (2 × 10^5^ cells/well) were also cultured in the absence of Raji cells in RPMI1640 complete media with and without lactic acid (30 mM) as negative and positive controls of apoptosis. The apoptotic cells were measured using the Annexin V-FITC (fluorescein isothiocyanate)/PI (propidium iodide) apoptosis detection kit (MBR, Iran). Briefly, after 48 h of incubation, the cells were harvested and centrifuged at 1500 rpm for 10^‘^. Cells were collected and counted and Annexin-V-FITC/PI labeling was carried out according to the instructions of the manufacturer (MBR, Iran). The cells were then stained with anti-CD3-APC (BioLegend). Finally, the cells were analyzed by flow cytometry (BD FACSCalibur, Biosciences, USA).

### Ros assay

ROS and superoxide detection assay kits (ab139476, USA) were used to evaluate the production level of intracellular ROS. T cells (2 × 10^5^ cells/well) cultured in RPMI1640 complete media with and without lactic acid (20 mM). After harvesting and washing, the cells were incubated with permeable green probe (reacts with hydroxyl radicals (HO), hydrogen peroxide, peroxynitrite (ONOO⎯), peroxyradical (ROO) and nitric oxide (NO)) and orange probe (in particular reacts with superoxide (O2 ⎯)) at 37 °C for 30^’^. The level of the antioxidant molecule GSH was measured with a GSH assay kit (ab112132, USA). After harvesting and washing, the cells were incubated at 24 °C with thiol green dye for 20 min. Finally, the cells were analyzed by flow cytometry. Based on the difference between the mean fluorescence intensity of lactic acid treated and untreated cells, the production of ROS/superoxide and GSH was determined.

### Real-time PCR

T cells (2 × 10^5^ cells/well) were cultured in RPMI1640 complete media with and without 20 mM lactic acid. According to the manufacturer’s instruction, total RNA was extracted from T cells using RNX-plus solution (RN7713C, Sinaclon, Iran) to determine levels of gene expression of NADPH oxidase subunit, gp91phox, and antioxidant enzymes including SOD1, SOD2, Nrf2, and CAT. Purity and concentration of RNA concentration were assessed using NanoDrop (Thermo Fisher). To eliminate genomic DNA the isolated RNA was treated with DNase I (Fermentas, USA). cDNA was then synthesized by a cDNA synthesis kit (Thermo Fisher Scientific, USA). Real-time PCR was performed using 2 × SYBR Green qPCR Mix plus (ROX) (Ampliqone, Denmark) on an ABI step one plus real-time PCR system (Applied Biosystem). Relative expression levels of these genes were normalized by 18 s rRNA as a housekeeping gene and calculated by the 2 − ΔΔCt method. The primers sequences are listed in the table S 1.

### Flow cytometric analysis

All samples were acquired and analyzed on a BD FACS Calibur (BD Biosciences, USA) with FlowJo software (v7.6.1). All experiments were conducted in triplicate and repeated three times.

### Statistical analysis

Statistical analysis was performed using Prism 7 software. Comparisons between treatment groups were conducted by independent t-test and ANOVA with Tukey’s post hoc test. Differences were accepted statistically significant when *P* < 0.05.

## Results

### Proliferation and cytokine secretion was suppressed by lactic acid

**Fig. 1:**
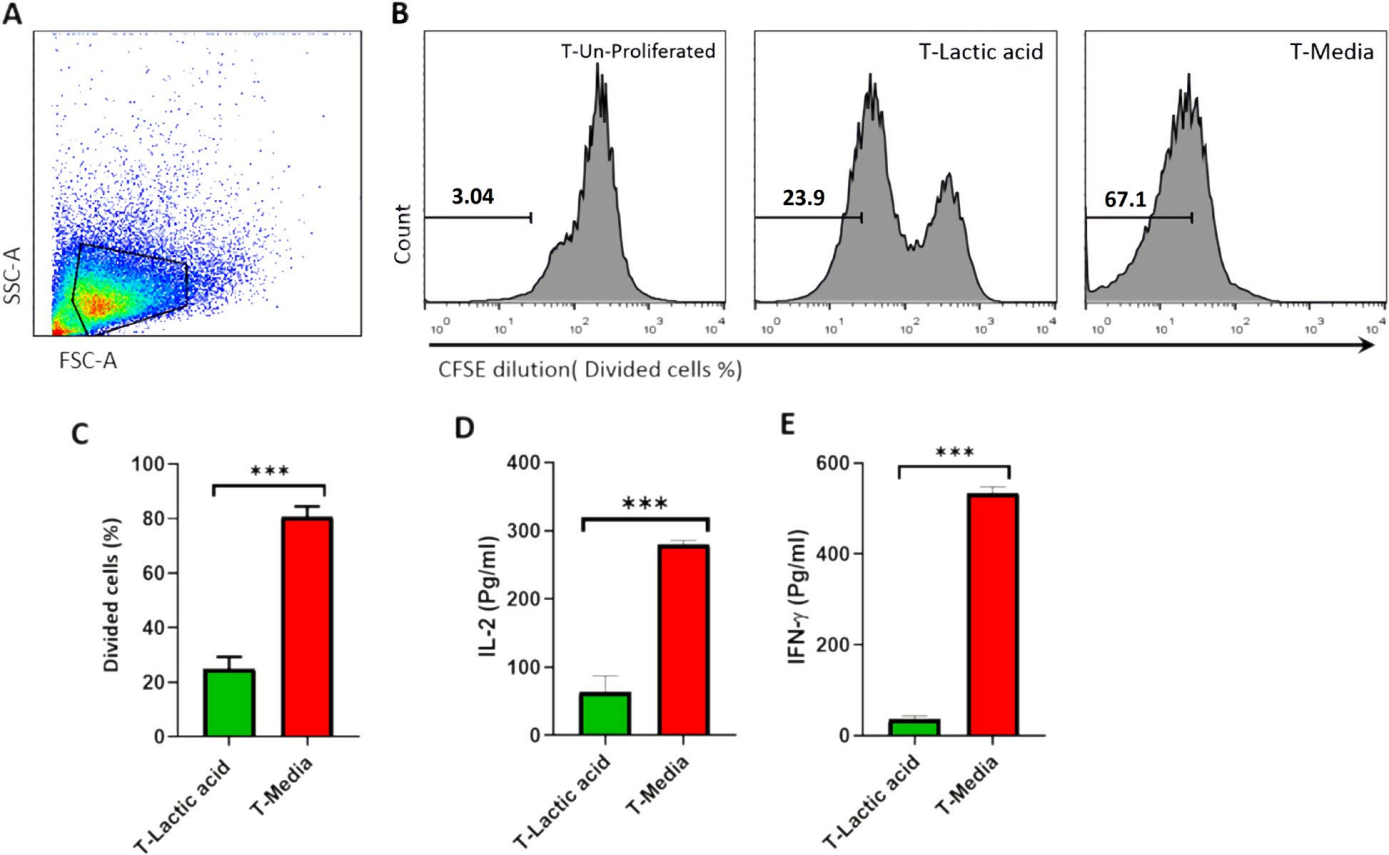
Lactic acid inhibited T cell Proliferation and cytokine secretion**.** Human T cells were labeled with CFSE dye, stimulated for 72 h with anti-CD3ε/CD28 antibody, and then cultured in media with or without lactic acid (20 mM). After 24 h the supernatants were collected and assessed for cytokine production by ELISA. Proliferation was analyzed by CFSE dilution through flow cytometry. (**A**) Representative gating strategies. Cells were gated on a forward vs. side scatter dot plot. (**B**) Histograms show the percentage of divided T cells. (**C**) The bar graph displays the average percentage of proliferated T cells in the presence or absence of lactic acid. (**D** and **E**) Bar graphs depict the concentrations of IL-2 and IFN-γ. Data are presented as mean ± SD from 3 separate experiments. An Independent T-test was used to examine the difference between the two groups. (***P < 0.001) IL: interleukin, CFSE: carboxyfluorescein succinimidyl ester, SD: standard deviation

We investigated the effect of lactic acid on various properties of T cells that have a role in T cell activation. A crucial characteristic of a successful immune response is T cell proliferation and cytokine secretion. We hypothesized that lactic acid has an immunosuppressive impact on the proliferation of T cells. To determine the effects of lactic acid we compared the suppressive impact of media containing lactic acid and media without lactic acid on the proliferation of stimulated human T cells (Fig. 1). The lactic acid concentration was set to 20 mM, which matches the lactic acid concentration experienced by T cells in previously published studies in human and murine tumors [20–22]. Representative flow cytometry data of the CFSE staining are presented in Fig. 1B. As expected, lactic acid treated T cells exhibited significantly lower rates of proliferation compared to the control group (Fig. 1C).

Next, we explored the effects of lactic acid on the production of T cell cytokines. T cells were stimulated with anti-CD3 and anti-CD28 antibodies and the level of IL-2 and IFN-γ was assessed by ELISA in the supernatants of cell culture after 24 h (Fig. 1D and E). We chose these cytokines due to IL-2 role in the growth, differentiation, and expansion of cytotoxic T lymphocytes (CTLs) and IFN-γ involvement in CTL proliferation and cytotoxic ability [23, 24]. Reductions in IL-2 and IFN-γ secretion were observed in T cells cultured with lactic acid. Finally, our data indicated that lactic acid inhibits the activation of T cells by limiting their proliferation and cytokine production.

### Lactic acid decreased ROS, superoxide and glutathione in T Cells

**Fig. 2:**
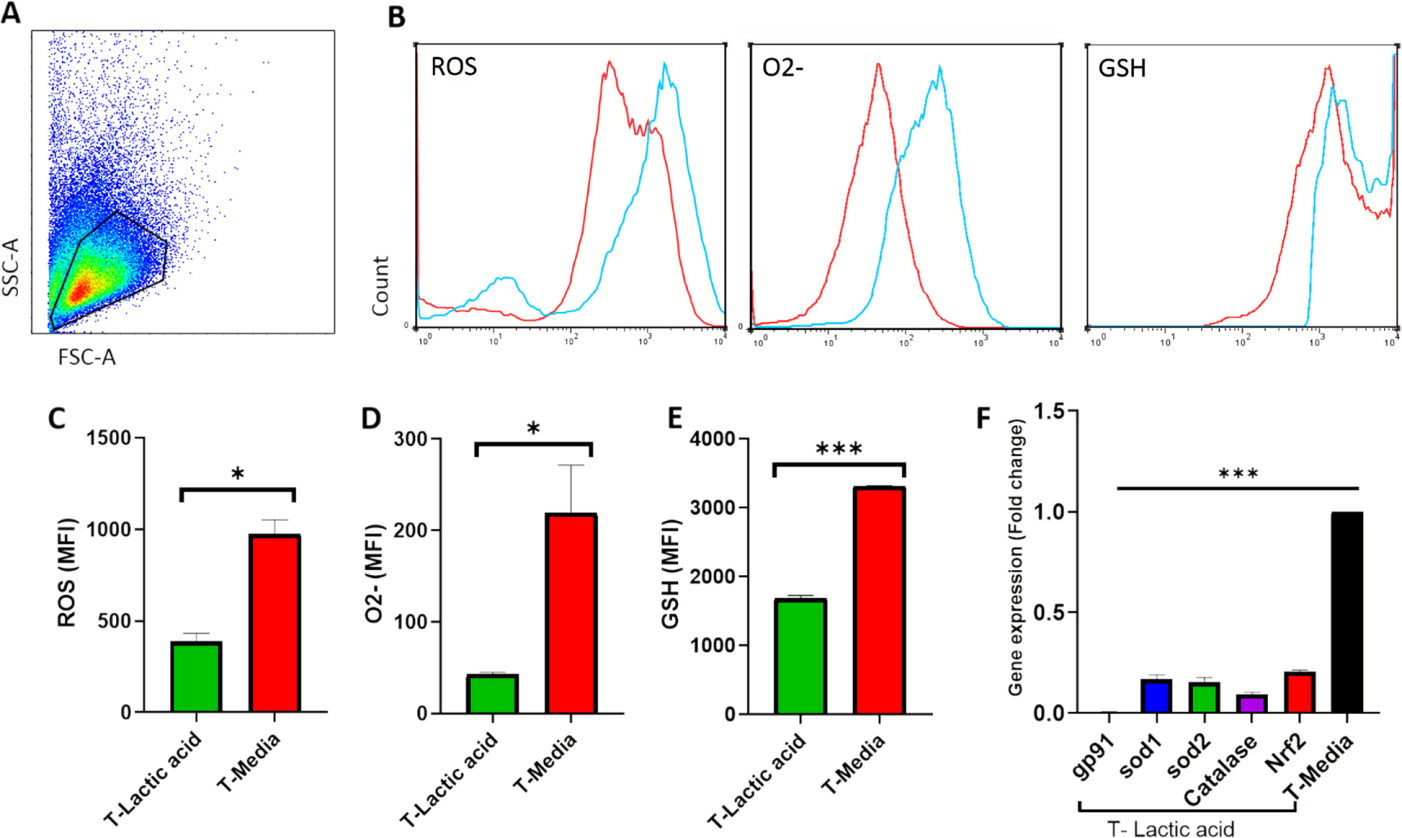
Lactic acid suppressed cellular redox system of T cells. T cells were cultured in media with or without lactic acid (20 mM). Cells were incubated with the green and orange probes that react with ROS and superoxide. To measure GSH, cells were incubated at 24 °C with thiol green dye. Finally, the cells were analyzed by flow cytometry. Based on the differences between the mean fluorescence intensity of lactic acid-treated and untreated cells, the production of ROS, superoxide, and GSH were determined. (**A**) Shows representative gating strategies for ROS, superoxide and GSH production in T cells. Cells were gated on a forward vs. side scatter dot plot. (**B**) Representative histograms displayed MFI of ROS, superoxide, and glutathione in untreated (blue line) and lactic acid-treated group (red line). Bar graphs show the production of ROS(**C**), Superoxide (**D**), and glutathione (**E**) in T cells. Data are presented as mean ± SD from a representative experiment (*n* = 3). An Independent T-test was used to examine the difference between the two groups. (**F**) Gene expression of the oxidant molecule NOX-gp91phox and the antioxidant molecules SOD1, SOD2, Nrf2, and CAT in lactic acid-treated and un-treated T cells were examined. Bar graphs show gene expression levels of (**a**) gp91phox, (**b**) CAT, (**c**) SOD1, (**d**) SOD2, and (**e**) Nrf2 in the lactic acid-treated T cells normalized to T-media group. (*P < 0.05, ***P < 0.001) GSH: glutathione, SOD: superoxide dismutase, CAT: catalase, MFI: mean fluorescence intensity, SD: standard deviation

We next asked how lactic acid may affect the cellular redox state of T cells as a factor responsible for activation of T cell receptor signaling and immune responses by T cells. To study oxidative stress in T cells we assessed the production of ROS, superoxide and intracellular levels of GSH in T cells cultured in media containing lactic acid (20 mM) (Fig. 2). At first, T cells were gated on a forward vs. side scatter dot plot (Fig. 2A). Then gated lymphocytes were analyzed for ROS, superoxide and GSH generation (Fig. 2B). Significant decreases in ROS and O_2_- production were seen in the lactic acid- treated T cells compared to the control group (Fig. 2C and D). We also observed that the intracellular levels of GSH were significantly lower in the T cells cultured with lactic acid compared to those cultured in plain media (Fig. 2E).

### Gene expression of antioxidant molecules decreased in T cells

We next studied the effects of lactic acid on T cell oxidative stress molecules and the balance between oxidants (NOX-gp91phox) and antioxidants (SOD1, SOD2, Nrf2, and CAT). Gene expression patterns in lactic acid treated T cells were also examined. There was a significant decrease in the expression of gp91phox in T cells cultured in lactic acid compared to that the control group (Fig. 2F). A similar trend was observed in gene expression of the antioxidant molecules SOD1, SOD2, Nrf2, and CAT and they were significantly reduced in lactic acid-treated cells compared to control T cells (Fig. 2F).

### DCA reduced lactic acid production of tumor cells

**Fig. 3:**
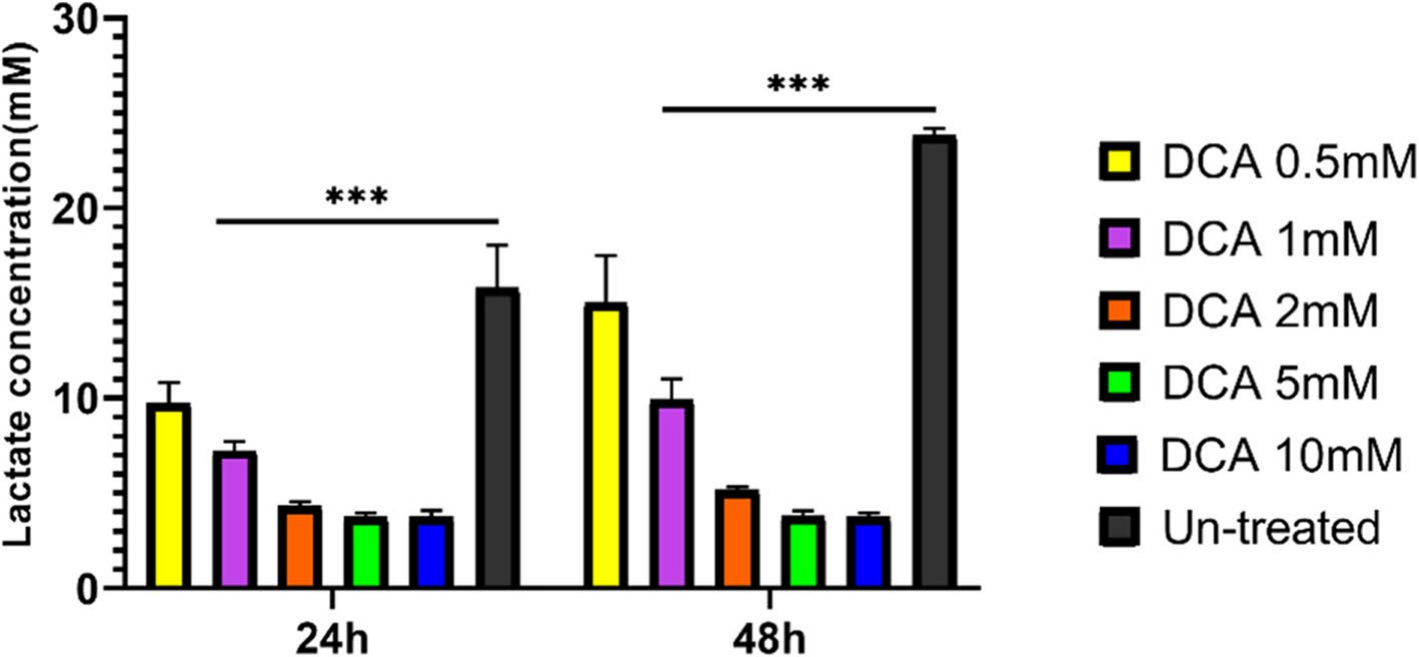
DCA reduced tumor-derived lactic acid. 2 × 10^5^ Raji cells were treated with various concentrations of DCA. Lactic acid concentrations in the tumor cell supernatants were measured after 24 h and 48 h. Lactic acid was measured using the colorimetric assay. Data are presented as mean ± SD from a representative experiment (*n* = 3). Two-way ANOVA was used to examine the difference between groups. Tukey’s post hoc test was used to compare means. (***P < 0.001) DCA: Dichloroacetate

High-lactic acid and low-glucose environments, such as seen in the tumor microenvironment, are immunosuppressive– particularly in the case of effector T cells. To manipulate tumor metabolic conditions and to overcome tumor immunosuppressive effects we used DCA to target the production of lactic acid in tumor cells. To assess the effects of DCA on lactic acid secretion of tumor cells we treated Raji cells with various concentrations of DCA and measured lactic acid concentrations in the supernatants of tumor cells after 24 h and 48 h. Two time-points were chosen because we speculated it takes time to observe DCA effects on the tumor cells. Our data shows DCA-treated tumor cells significantly decreased lactic acid production of lymphoma cells in comparison to untreated cells (Fig. 3). We also found that DCA had suppressive effects within 24 h and treating cells for 48 h is not required, in addition, higher dosages of DCA increased the suppressive effects. Doses higher than 2 mM showed no further reduction in lactic acid levels as they were toxic to the cells. We thus chose a dose of 1 mM DCA to continue our study. At this dose the tumor cells appeared healthy and their ability to produce lactic acid was reduced, supporting our conclusion that DCA inhibits lactic acid production by tumor cells.

### Treating tumor cells with DCA improves the proliferation capacity of T cells

**Fig. 4:**
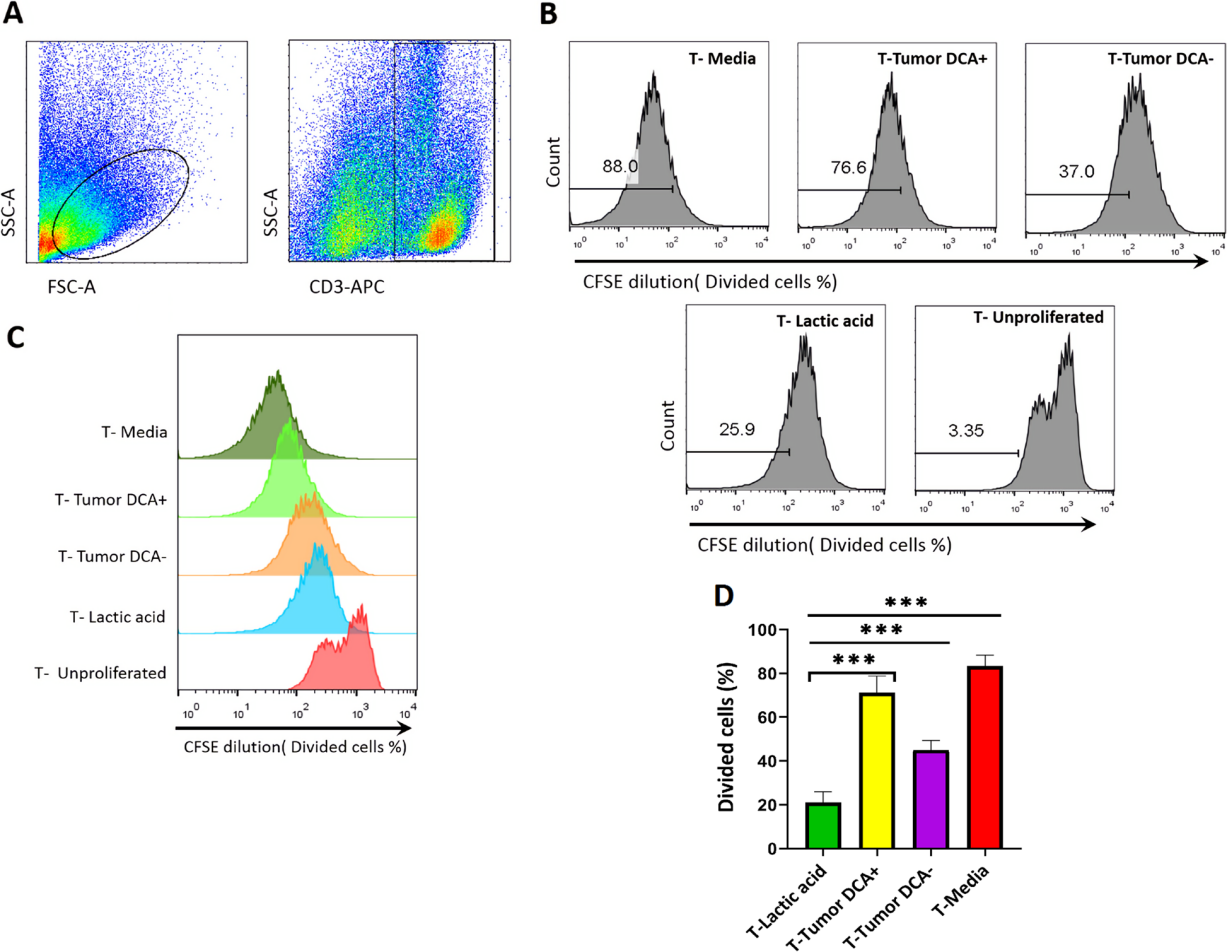
DCA enhances proliferation capacities of T cells. 2 × 10^5^ mitomycin C-treated Raji cells were cultured in the presence or absence of DCA (1 mM) for 24 h. After removal of DCA, CFSE-labeled T cells were co-cultured with DCA treated-Raji cell at a 1:1 ratio for 72 h in the presence of anti-CD3ε/CD28 antibody. Anti-CD3 staining was used to differentiate T cells from tumor cells. CFSE dilution was used as an indicator of cell proliferation. (**A**) Representative gating strategy. Cells were gated on a forward vs. side scatter dot plot and CD3 positive cells were gated. (**B**) Histograms depict the percentage of divided T cells in five groups. (**C**) Overlaid histogram CFSE labeled T cells. (**D**) The bar graph displays the average percentage of proliferated T cells in different conditions. Data are presented as mean ± SD from 3 different experiments. On e-way ANOVA was used to examine the difference between groups. Tukey’s post hoc test was used to compare means. (***P < 0.001) IL: interleukin, CFSE: carboxyfluorescein succinimidyl ester, SD: standard deviation. Simple lines above the bars in the bar graph show comparisons between multiple groups, for example, the line above the T-media is depicting a comparison between the T-media group and all other groups, but the capped line shows a comparison between certain two groups

To examine the function of the T cells co-cultured with tumor cells with regard to reduce lactic acid production we explored the proliferative response of T cells after they were exposed to lactic acid-inhibited tumor cells. T cells were stimulated with anti-CD3 and CD28 antibodies, labeled with CFSE dye and then co-cultured with equivalent numbers of DCA treated and untreated Raji cells for 72 h. T cells were also cultured in the presence and absence of lactic acid (20 mM) as a positive and negative control for lactic acid (Fig. 4). Representative flow cytometry data of the CFSE staining are presented in Fig. 4B and C. As expected DCA significantly decreased the suppressive effect of lactic acid secreted by lymphoma cells on the T cell proliferative responses (Fig. 4D). However the DCA-treated group did not proliferate as well as those cultured in plain media but the rate of proliferation by the DCA-treated group was increased by 25% compared to the DCA-negative group. Raji cells were also treated with mitomycin C for 30^’^ to prevent proliferation. We were concerned whether mitomycin C could change the glucose metabolism of tumor cells and dampen their lactic acid secretion. To address this we compared the concentrations of lactic acid in the supernatants of mitomycin C-treated and untreated-Raji cells. The data displayed no difference in the lactic acid production between the two groups (figure S 4).

### Increased T cell cytokine secretion by inhibition of lymphoma cells lactic acid production

**Fig. 5:**
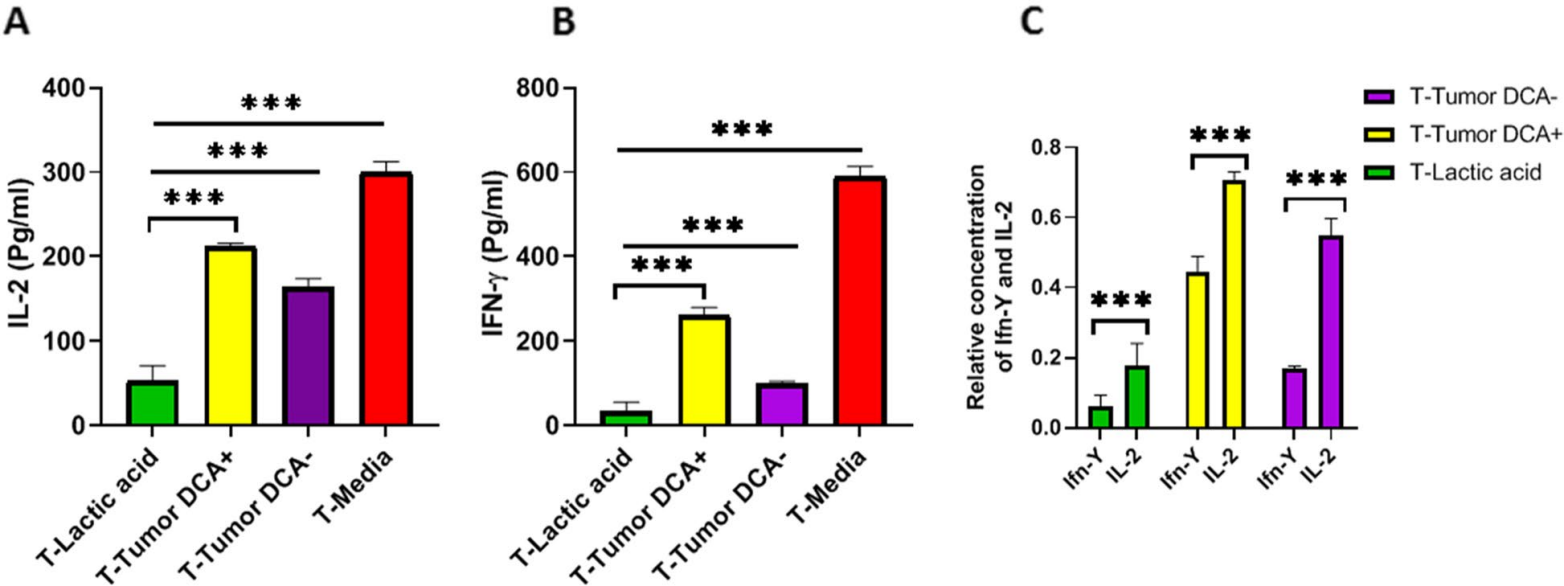
DCA elicits enhanced T cell cytokine secretion. 2 × 10^5^ T cells were co-cultured at a 1:1 ratio with DCA treated Raji cells. The supernatants were collected after 24 h and the levels of IFN-γ (**A**) and IL-2 (**B**) were evaluated by ELISA. (**C**) IFN-γ and IL-2 levels normalized to the T-Media group. Data are presented as mean ± SD from 3 different experiments. One-way ANOVA was used to examine the difference between groups. Tukey’s post hoc test was used to compare means. (***P < 0.001) IL: interleukin, SD: standard deviation. Simple lines above the bars in the bar graph show comparisons between multiple groups, but the capped line shows a comparison between certain two groups

Different groups of T cells were evaluated for cytokine production. ELISAs were used to examine IL-2 and IFN-γ concentrations.We examined cytokine concentrations in the supernatants of T cells co-cultured with DCA-treated Raji cells (fig. 5). DCA treatment led to enhanced production of IFN-γ, and IL-2 by T cells compared to the DCA untreated group (fig. 5A and B). Consistent with our previous findings DCA could not rescue cytokine production as well as the T cell media-only group. Our results showed that IFN-γ secretion is more vulnerable to lactic acid compared to IL-2 (fig. 5C). Lactic acid secreted by tumor cells in the DCA-negative group suppressed IFN-γ secretion nearly 3 times more than IL-2 and DCA can nearly doubles the IFN-g secretion and decrease the IL-2/IFN-g ratio to 1.5.

### DCA rescued T cells from lactic acid

**Fig. 6:**
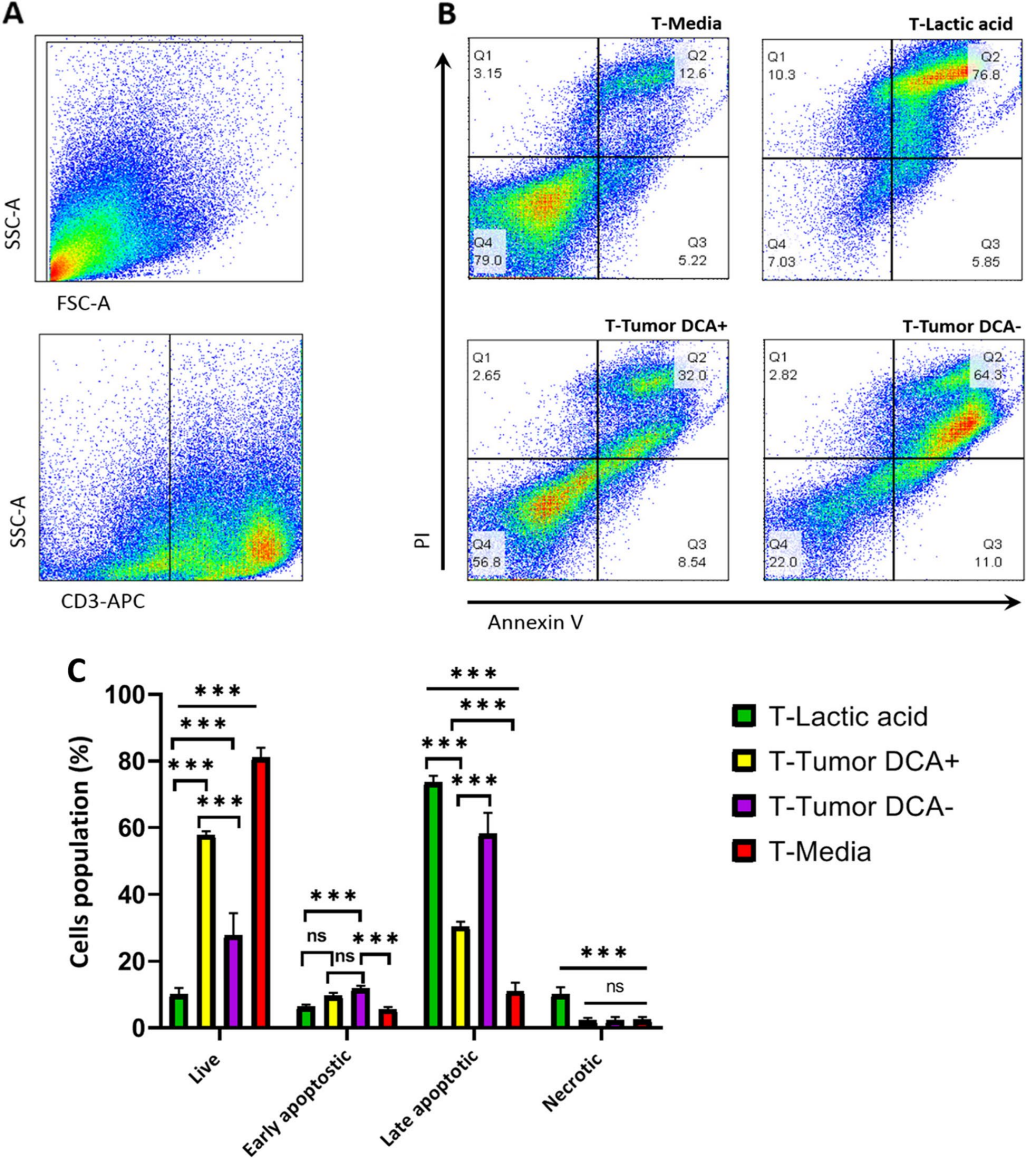
DCA increased T cell viability. 2 × 10^5^ DCA-treated Raji cells were co-cultured with T cells at 1:1 ratios for 48 h. The apoptotic cells were measured using the annexin V/PI apoptosis detection kit. Anti-CD3 staining was used to differentiate T cells from tumor cells. (**A**) Representative gating strategy. Cells were gated on a forward vs. side scatter dot plot and CD3-positive cells were gated. (**B**) Flow cytometry density plots representing annexin V (X-axis) and PI (Y-axis) staining of T cells. Annexin V-positive and PI-negative staining indicating early apoptosis. Both the annexin V and PI-positive populations show late apoptosis, annexin V-negative and PI-positive population depict necrosis, both the annexin V and PI-negative staining indicating live cells. (**C**) Bar graphs display the average percentage of 4 groups of cells populations in different conditions (Mean ± SD of three assays). Two-way ANOVA was used to examine the difference between groups. Tukey’s post hoc test was used to compare means. (***P < 0.001) ns: Not significant, PI: propidium iodide, SD: standard deviation. Simple lines above the bars in the bar graph show comparisons between multiple groups, but the capped line shows a comparison between certain two groups

For evaluation of apoptosis induced by tumor-derived lactic acid we explored the exposure of cell surface phosphatidylserine using Annexin V/PI double staining (Fig. 6). Representative results of flow cytometry data of the Annexin V/PI staining are presented in Fig. 6B. T cells cultured with lactic acid (30 mM) served as a positive control for apoptosis because this concentration of lactic acid was cytotoxic for T cells [unpublished]. Flow cytometry analyses revealed a significant positive effect for DCA on T cell viability. The percentage of viable T cells in the DCA-treated group was nearly twice that in the DCA-untreated group (Fig. 6C). The Annexin V/PI assay indicated that lactic acid induced late-stage apoptosis in T cells and the difference of late apoptotic and necrotic cells was not significant between the groups.

## Discussion

A positive correlation has been reported between high levels of lactic acid and tumor progression in a variety of tumors [22, 25]. The relationship between lactic acid levels in the tumor microenvironment and T cell activation is a new concept in this context [9]. A significant decline in CTL cytolytic activity was observed in the low PH of the Tumor microenvironment in the tumor-bearing mice [26, 27]. In our study, we found that lactic acid decreased the function of T cells in vitro and it has an immunosuppressive impact on the proliferation of T cells. Prior studies have shown the inhibiting effect of lactic acid on effector T cell proliferation [28]. One of the known mechanisms that tumor cells utilize to limit T cell proliferation is lactic acid elevation which results in the blocking of glyceraldehyde 3-phosphate dehydrogenase (GAPDH) and 3-phosphoglycerate dehydrogenase, leading to the depletion of subsequent glycolytic intermediates including the 3-phosphoglycerate derivative serine which is known to be an essential factor for proliferation of T cells [20]. We have observed that lactic acid could also impair the IL-2 and IFN-γ secretion of T cells. Consistent with previous findings it has been shown that lactic acid reduces IL-2, IFN-γ, and granzyme B expression in human T cells [29]. Brand et al. proposed that that intracellular acidification restricts NFAT regulation, a significant transcription factor involved in IFN-γ transcriptional control. Besides, acidification can also disrupt the translocation of NFAT to the nucleus [9, 30].

We assessed oxidative stress in T cells because it was previously shown that ROS perform as messengers for T cell receptor signaling in the steady-state and upon antigen recognition. Therefore, ROS play a critical role in T cell activation [31–33]. Here we investigated the production of ROS, superoxide and intracellular levels of GSH in T cells treated with lactic acid. We also examined gene expression of NOX-gp91phox as an oxidant molecule and SOD1, SOD2, Nrf2 and CAT as antioxidants. Since under normal conditions the levels of endogenous ROS are tightly regulated by different antioxidant systems inside the cell [34]. Significantly lower production of both oxidants and antioxidants was seen in the lactic acid-treated T cells. The levels of gene expression paralleled this. Reduced ROS levels result in deficient signaling, which results in low activation and proliferation [12]. Also, GSH is essential for T cell effector functions through its regulation of metabolic activity. Mice with GSH-deficient T cells showed restricted antiviral responses in vivo [14]. Therefore, inhibition of ROS and GSH is maybe one of the ways that lactic acid restricts T cell activation. Our observations were not supported in a recent report showing a rapid and striking elevation of intracellular ROS which was caused by the exposure of activated CD4 + T cells to lactate [35]. However in that report the levels of ROS in T cells were measured in the presence of 10 mM of sodium lactate at three-time points. They assessed ROS at 5, 10 and 30^’’^ after the exposure of T cells to lactate. The levels of the ROS showed a downward trend from the first-time point to the third-time point. We investigated the amount of oxidant and antioxidant molecules after culturing T cells for 24 h in the presence of 20 mM of lactic acid. The duration of our test and lactic acid concentrations were different from those in the recent study [35]. The mechanism(s) through which lactic acid interrupts the redox system of T cells remains for future research.

A promising therapeutic strategy is to target the glycolysis pathway of tumor cells as the impairment of glucose metabolism could cause defects in tumor cells growth and survival [36, 37] It further decreases their lactic acid secretion and acidification of the tumor microenvironment that impairs the T and NK cells' anti-tumor immune responses [9, 27, 38]. Consequently, reducing the amounts of intratumoral lactic acid and acidification improves immunosurveillance potentially the effectiveness of cancer immunotherapies [15, 39–41].

In recent years DCA which already is used for the treatment of lactic acidosis has been considered as an anticancer agent [42, 43]. DCA targets cancer cells and inhibits pyruvate dehydrogenase kinase, the inhibitor of pyruvate dehydrogenase. Therefore, DCA alters the metabolism of tumors from glycolysis towards oxidative phosphorylation [44]. Activation of PDH induces pyruvate mitochondrial oxidation and limits the metabolic advantage of tumor cells. Besides, DCA could prevent acidosis in the tumor microenvironment by decreasing lactic acid secretion and thus leading to inhibition of tumor growth [45, 46]. The direct effects of DCA on cancer cells have been tested in most studies to date but here we have focused on evaluating the effects of DCA on tumor-derived lactic acid and its impact on T cells. Our results indicate that DCA can restore the T cell proliferative response and cytokine production from the suppressive effect of tumor-derived lactic acid. Interestingly, we observed the proliferation of T cells that were co-cultured with untreated tumor cells was significantly higher than rate of proliferation in the lactic acid group. Surprisingly, the lactic acid concentration in untreated tumor cells were greater than lactic acid group (i.e. 20 mM). It is illustrated that low concentrations of lactic acid are not detrimental to T cell function and are even beneficial [47–50]. We supposed because tumor cells produce lactate gradually, initially, T cells promote their function by utilizing lactate. But lactic acid concentration raises over time and finally disrupts T cells function. In contrast, in the “control group” (i.e. T- cells with lactic acid), sudden exposure of T cells to a high concentration of lactic acid is quite damaging for them. DCA also reduced apoptosis in T cells and preserved their viability. These data are in the line with the previous study in which diclofenac promoted anti-tumor response of T cell by reprogramming tumor glycolysis and inhibiting their lactic acid production [41]. Activation, viability, and effector functions of T cells were maintained in vitro following diclofenac treatment. They also showed that treatment of tumor cells with diclofenac caused an increase the in vitro anti- PD-1-mediated T cell killing of tumor cells. Diclofenac also enhanced the response to the anti-PD-1 blockade in tumor-bearing mice [41], as there was a negative correlation between response to anti-PD-1 therapy and metabolic genes overexpression [51]. To better understand the impact of DCA on checkpoint therapy we suggest further studies on using the combination of DCA and immune checkpoint inhibitors to treat tumors are warranted.

## Conclusion

Taken together our results suggest that the accumulation of lactic acid in the tumor microenvironment restricts T cell responses and could interfere with the success of T cell therapy. For this reason, blocking microenvironment acidification prior to immunotherapy could strengthen the anti-tumor responses. It has been shown that DCA supports anti-tumor responses of T cells by metabolic reprogramming of tumor cells. DCA reduced the lactic acid production of tumor cells and preserved T cell activation. Tumor metabolic alteration illustrates a promising strategy to develop novel immunotherapies or improve the existing ones. Our future research will be to evaluate DCA therapy in combination with adoptive cellular therapy beginning with murine models in vivo.

## Supplementary Information

**Additional file 1: Supplementary figure 1.** pH of media culture with lactic acid. Different concentration of lactic acid were added to RPMI 1640 medium supplemented with 10% FBS. The pH were measured with pH meter. **Supplementary figure 2.** The purity of T cells is represented. PBMCs were seeded into 24-well plates (1.5×106 cells/well) and cultured in RPMI1640 containing 10%FBS and 100IU hIL-2. To activate and enrich T cells, PBMCs were cultured with 3 ug/ml anti-CD3 antibody and 10 ug/ml anti-CD28 antibody. After 4 days of incubation at 37°C purity of T cells was assayed by flow cytometry using APC conjugated anti-human CD3. **Supplementary figure 3.** Viability of raji cells were treated with different concentration of DCA. 2 × 10^5^ Raji cells were treated with various concentrations of DCA. The apoptosis was detected by flow cytometry using the PI staining. DCA: dichloroacetate PI: propidium iodide. **Supplementary figure 4. **DCA reduced tumor-derived lactate. 2 × 10^5^ Raji cells were treated in the presence or absence of mitomycin C. Lactate concentration in the tumor cells supernatant was measure after 24h and 48h. Lactate was measured using the colorimetric assay. Data are presented as mean ± SD from a representative experiment (*n* = 3). Two-way ANOVA was used to examine the difference between groups. Tukey’s post hoc test was used to compare means. **Supplementary table 1.** Primers used for gene expression analysis through real-time PCR.

## Data Availability

The datasets generated and analyzed during the current study are available from the corresponding author on reasonable request.

## Abbreviations

DCA: Dichloroacetate
ACT: Adoptive cellular therapy
CAR: Chimeric antigen receptor
ROS: Reactive oxygen species
IL: Interleukin
CFSE: Carboxyfluorescein succinimidyl ester
GSH: Glutathione
SOD: Superoxide dismutase
CAT: Catalase
MFI: Mean fluorescence intensity
PI: Propidium iodide
