# Supplementary material for: Restricting tumor lactic acid metabolism using dichloroacetate improves T cell functions
Source: BMC Cancer. 2022 Jan 6;22:39. doi: 10.1186/s12885-021-09151-2 (PMC8734242; doi:10.1186/s12885-021-09151-2)
Supplement: Supplementary file 1 — Additional file 1: Supplementary figure 1. pH of media culture with lactic acid. Different concentration of lactic acid were added to RPMI 1640 medium supplemented with 10% FBS. The pH were measured with pH meter. Supplementary figure 2. The purity of T cells is represented. PBMCs were seeded into 24-well plates (1.5×10^6^ cells/well) and cultured in RPMI1640 containing 10%FBS and 100IU hIL-2. To activate and enrich T cells, PBMCs were cultured with 3 ug/ml anti-CD3 antibody and 10 ug/ml anti-CD28 antibody. After 4 days of incubation at 37°C purity of T cells was assayed by flow cytometry using APC conjugated anti-human CD3. Supplementary figure 3. Viability of raji cells were treated with different concentration of DCA. 2 × 10^5^ Raji cells were treated with various concentrations of DCA. The apoptosis was detected by flow cytometry using the PI staining. DCA: dichloroacetate PI: propidium iodide. Supplementary figure 4. DCA reduced tumor-derived lactate. 2 × 10^5^ Raji cells were treated in the presence or absence of mitomycin C. Lactate concentration in the tumor cells supernatant was measure after 24h and 48h. Lactate was measured using the colorimetric assay. Data are presented as mean ± SD from a representative experiment (n = 3). Two-way ANOVA was used to examine the difference between groups. Tukey’s post hoc test was used to compare means. Supplementary table 1. Primers used for gene expression analysis through real-time PCR. [file 12885_2021_9151_MOESM1_ESM.docx]

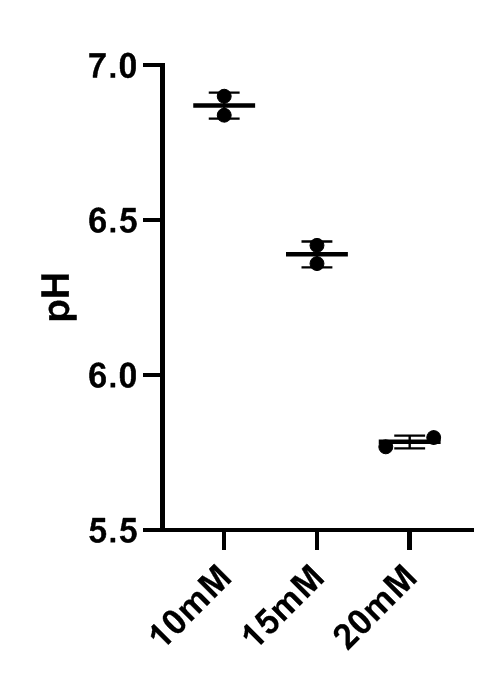
**Supplementary Data**

**Supplementary figure 1.** pH of media culture with lactic acid. Different concentration of lactic acid were added to RPMI 1640 medium supplemented with 10% FBS. The pH were measured with pH meter.

Unstained Stained

**Count**

**APC anti-human CD3**


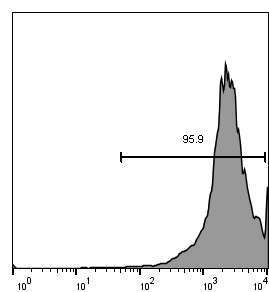

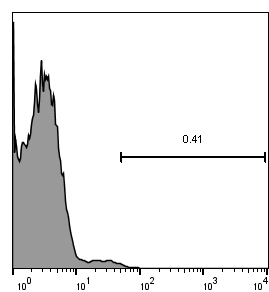


**Supplementary figure 2.** The purity of T cells is represented. PBMCs were seeded into 24-well plates (1.5×106 cells/well) and cultured in RPMI1640 containing 10%FBS and 100IU hIL-2. To activate and enrich T cells, PBMCs were cultured with 3 ug/ml anti-CD3 antibody and 10 ug/ml anti-CD28 antibody. After 4 days of incubation at 37°C purity of T cells was assayed by flow cytometry using APC conjugated anti-human CD3.


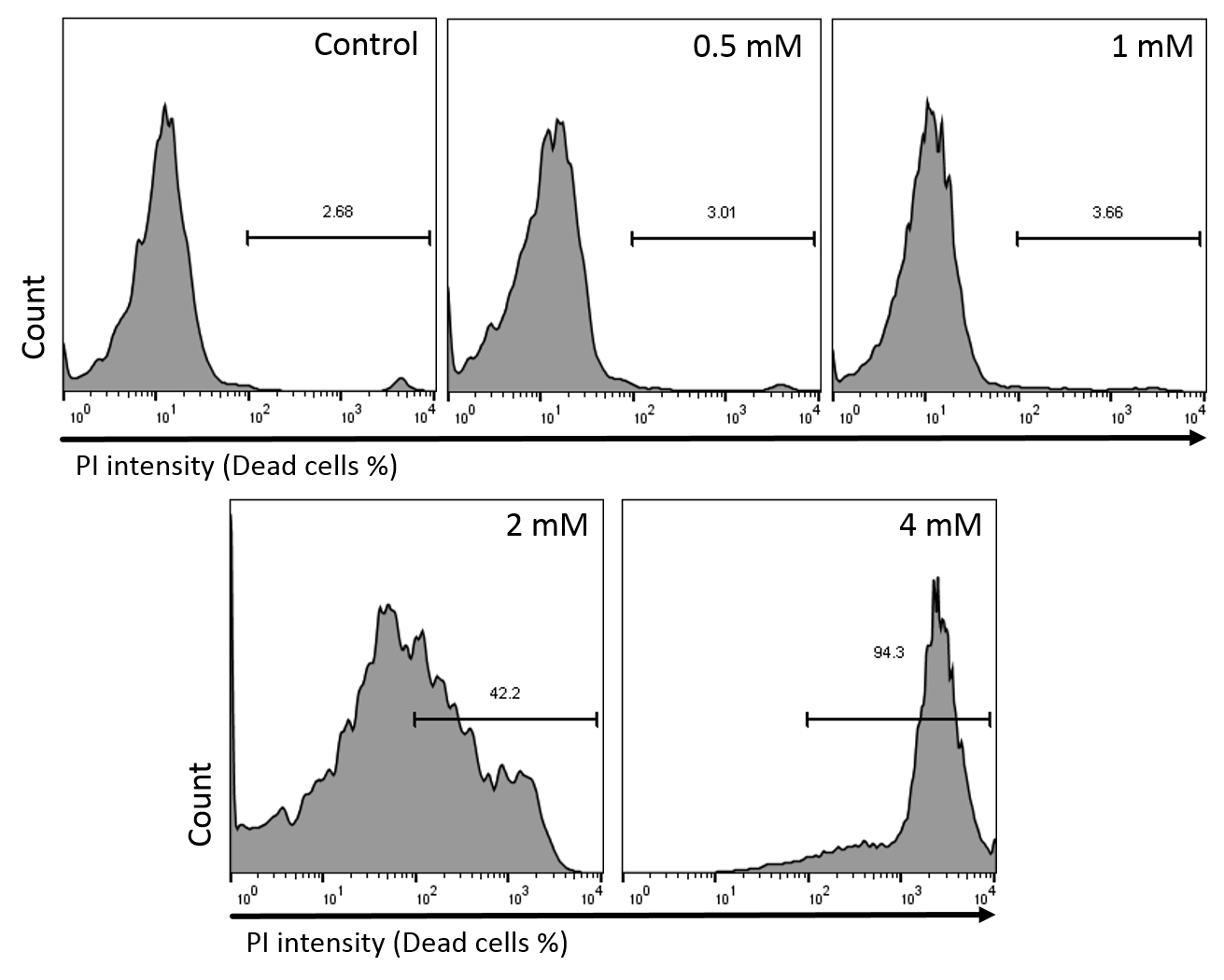


**Supplementary figure 3.** Viability of raji cells were treated with different concentration of DCA. 2 × 10^5^ Raji cells were treated with various concentrations of DCA. The apoptosis was detected by flow cytometry using the PI staining. DCA: dichloroacetate PI: propidium iodide


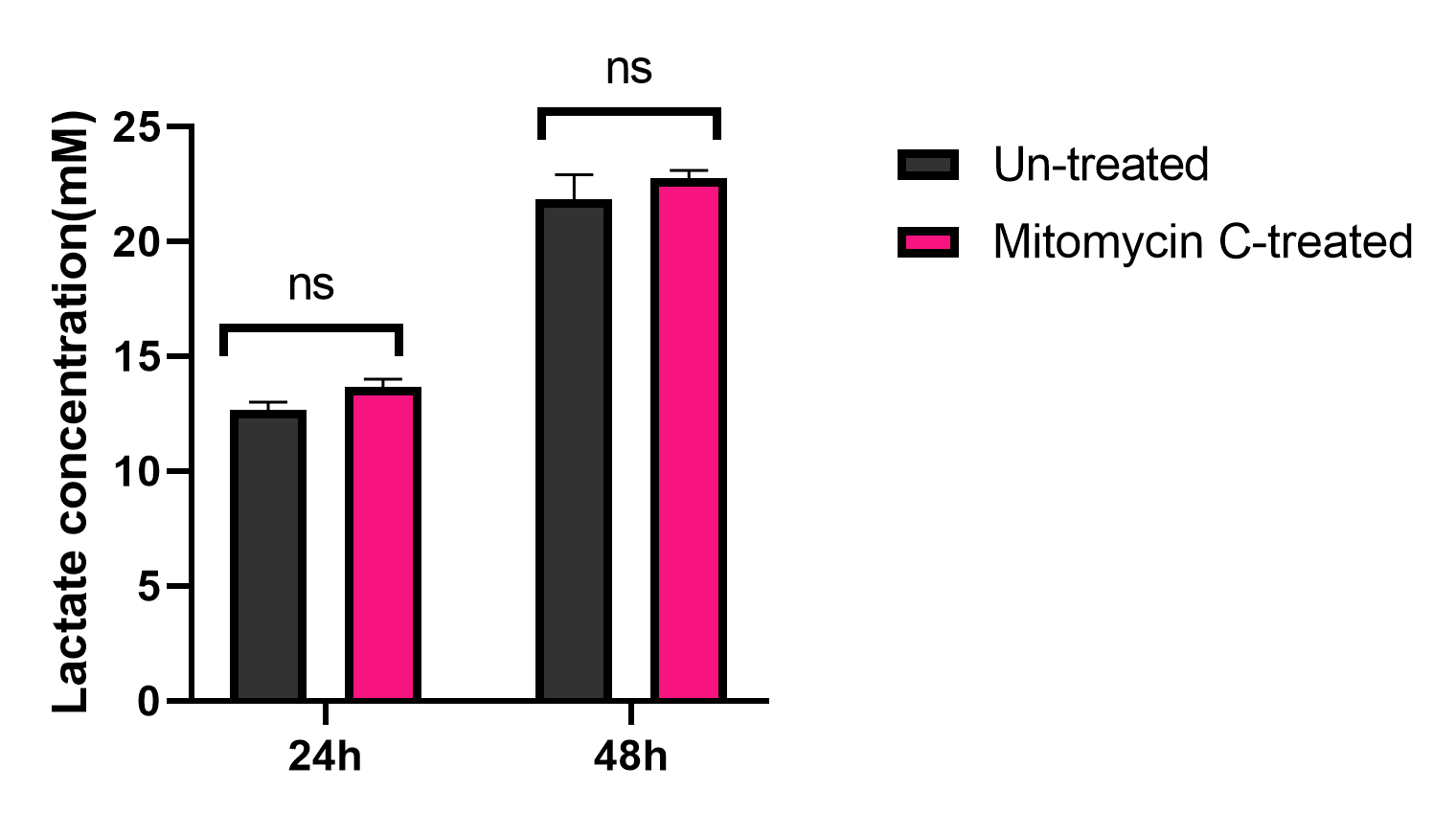


**Supplementary figure 4.** DCA reduced tumor-derived lactate. 2 × 10^5^ Raji cells were treated in the presence or absence of mitomycin C. Lactate concentration in the tumor cells supernatant was measure after 24h and 48h. Lactate was measured using the colorimetric assay. Data are presented as mean ± SD from a representative experiment (n = 3). Two-way ANOVA was used to examine the difference between groups. Tukey’s post hoc test was used to compare means.

| **Gene name** | **Forward primer (5′→ 3′)** | **Reverse primer (5′→ 3′)** |
| --- | --- | --- |
| **Gp91 phox** | CTGGAAACCCTCCTATGACTTG | GTGATGACCACCTTCTGTTGAG |
| **CAT** | TGCTGAATGAGGAACAGAGGAA | CCTCACAGATTTGCCTTCTCC |
| **Nrf-2** | CCATTCCTGAGTTACAGTGTCT | CTGTGGAGAGGATGCTGC |
| **SOD1** | AGCGAGTTATGGCGACGAAG | CAGCCTGCTGTATTATCTCCA |
| **SOD2** | CTCAGGTTGGGGTTGGCT | TGAAGGTAGTAAGCGTGCTCC |
| **18s rRNA** | GTAACCCGTTGAACCCCATT | CCATCCAATCGGTAGTAGCG |

**Supplementary table 1:** Primers used for gene expression analysis through real-time PCR.
